# Supplementary material for: Preventive Behaviors and Information Sources during COVID-19 Pandemic: A Cross-Sectional Study in Japan
Source: Int J Environ Res Public Health. 2022 Nov 4;19(21):14511. doi: 10.3390/ijerph192114511 (PMC9658992; doi:10.3390/ijerph192114511)
Supplement: Supplementary file 1 [file ijerph-19-14511-s001.zip › ijerph-1970186-supplementary.pdf]

International Journal of
Environmental Research and Public Health

MDPI

Supplementary Materials

Table S1. Factors associated with the COVID-19 preventive measures.

	**OR (95% CI)**		
	**3Cs**	**Hand hygiene**	**Respiratory hygiene**
Age (SA)			
20's	1.00	1.00	1.00
30's	1.41(1.25-1.60)***	1.44(1.27-1.63)***	1.55(1.39-1.73)***
40's	1.59(1.41-1.80)***	1.45(1.28-1.65)***	1.84(1.64-2.05)***
50's	2.05(1.81-2.33)***	1.64(1.43-1.89)***	2.35(2.09-2.66)***
60's	2.21(1.94-2.51)***	1.71(1.47-1.98)***	2.51(2.22-2.84)***
70's+	2.26(1.97-2.60)***	1.78(1.51-2.11)***	2.25(1.96-2.58)***
Gender (SA)			
Female	1.00	1.00	1.00
Male	0.71(0.66-0.76)***	0.65(0.60-0.71)***	0.61(0.57-0.65)***
Highest educational level (SA)			
Middle and High school	1.00	1.00	1.00
Junior college	1.06(0.99-1.14)	1.10(1.00-1.22)	1.14(1.05-1.24)**
University	1.05(0.98-1.12)	0.97(0.90-1.06)	1.02(0.96-1.09)
Graduate School	1.11(0.97-1.28)	0.81(0.69-0.95)*	0.88(0.76-1.01)
Region (SA)			
Hokkaido and Tohoku	1.00	1.00	1.00
Kanto	1.34(1.23-1.47)***	1.08(0.96-1.21)	1.18(1.07-1.30)**
Chubu	1.03(0.93-1.14)	0.86(0.75-0.98)*	1.08(0.97-1.19)
Kinki	1.15(1.04-1.27)**	0.93(0.81-1.05)	1.13(1.02-1.25)*
Chugoku and Shikoku	1.01(0.90-1.14)	0.72(0.62-0.83)***	0.97(0.86-1.10)
Kyushu	1.07(0.96-1.19)	0.87(0.75-1.00)*	1.17(1.05-1.31)**
Occupation type (SA)			
Agriculture, forestry and fisheries	1.00	1.00	1.00
Construction	1.19(0.81-1.74)	1.04(0.69-1.56)	1.30(0.91-1.86)
Manufacturing	1.22(0.86-1.75)	1.14(0.78-1.67)	1.32(0.94-1.84)
Information and communications	1.16(0.79-1.70)	1.23(0.82-1.86)	1.42(0.99-2.03)
Transportation and postal services	1.01(0.69-1.49)	1.42(0.94-2.15)	1.34(0.94-1.92)
Wholesale and retail trade	1.15(0.80-1.64)	1.27(0.86-1.88)	1.35(0.96-1.89)
Finance and insurance	1.24(0.84-1.82)	1.33(0.87-2.04)	1.36(0.94-1.96)
Real estate and goods rental and leasing	1.02(0.68-1.53)	0.95(0.60-1.48)	1.29(0.87-1.92)
Scientific research, professional and technical services	1.39(0.93-2.10)	1.53(0.95-2.46)	1.57(1.05-2.35)*
Accommodations, food and beverage services	1.13(0.77-1.67)	1.18(0.77-1.81)	1.25(0.86-1.81)
Living-related and personal services and amusement services	1.06(0.71-1.59)	1.46(0.92-2.32)	1.27(0.86-1.87)
Education and learning support	1.11(0.77-1.62)	1.28(0.84-1.94)	1.34(0.94-1.92)
Medical healthcare and welfare	1.08(0.75-1.55)	1.16(0.78-1.72)	1.22(0.87-1.72)

Combined services	1.23(0.78-1.93)	1.07(0.65-1.78)	1.38(0.89-2.14)
Services (not elsewhere classified)	1.20(0.84-1.72)	1.26(0.86-1.86)	1.35(0.97-1.89)
Public service (not elsewhere classified)	0.98(0.67-1.43)	1.20(0.79-1.81)	1.58(1.10-2.26)*
Students	0.95(0.63-1.45)	1.10(0.72-1.70)	1.07(0.73-1.56)
Homemaker	1.49(1.05-2.12)*	1.58(1.07-2.32)*	1.50(1.08-2.10)*
Others	1.27(0.90-1.80)	1.21(0.83-1.76)	1.11(0.80-1.54)
Annual household income in 2020 (million JPY) (SA)			1.01(1.00-1.03)
Household size including respondent (SA)			
1	1.00	1.00	1.00
2	1.21(1.10-1.32)***	1.35(1.21-1.51)***	1.18(1.08-1.30)**
3	1.08(0.98-1.18)	1.33(1.19-1.48)***	1.07(0.97-1.18)
4	0.99(0.88-1.10)	1.27(1.12-1.44)***	1.16(1.04-1.30)*
5	1.05(0.90-1.22)	1.42(1.19-1.71)***	1.24(1.06-1.45)**
More than 6	0.96(0.78-1.18)	1.22(0.95-1.57)	1.12(0.91-1.39)
Marital size (SA)			
Married (including de facto marriage)	1.00	1.00	1.00
Not married (without partner)	1.11(1.02-1.21)*	1.14(1.03-1.27)*	1.20(1.10-1.31)***
Not married (with a partner)	1.02(0.89-1.17)	1.20(1.02-1.40)*	1.21(1.06-1.38)**
Widowed	1.02(0.89-1.17)	1.05(0.85-1.30)	1.05(0.90-1.24)
Divorced	1.07(0.96-1.20)	1.18(1.01-1.37)*	1.17(1.03-1.33)*
Self-reported health status (SA)			
Very good	1.00	1.00	1.00
Good	0.91(0.85-0.97)**	1.01(0.92-1.11)	1.01(0.94-1.09)
Fair	0.89(0.83-0.96)**	0.95(0.86-1.04)	0.95(0.88-1.03)
Poor	0.87(0.79-0.97)**	0.98(0.86-1.12)	0.92(0.83-1.03)
Very poor	0.98(0.81-1.18)	0.82(0.65-1.02)	0.83(0.68-1.00)*
How confident are you filling out medical forms by yourself?" (health literacy) (SA)			
Not at all	1.00	1.00	1.00
A little bit	0.93(0.70-1.23)	1.56(1.18-2.05)**	1.20(0.93-1.55)
Somewhat	1.08(0.84-1.40)	1.71(1.33-2.19)***	1.25(0.99-1.58)
Extremely	1.30(1.01-1.67)*	2.28(1.78-2.92)***	1.52(1.21-1.91)***
Quite a bit	1.58(1.23-2.04)***	2.63(2.05-3.38)***	1.86(1.48-2.35)***
Have you ever received a COVID-19 test? (MA)			
No		2.18(1.75-2.72)***	
Yes – PCR test		1.78(1.39-2.27)***	
Yes – antigen test		1.55(1.11-2.18)*	0.74(0.58-0.96)*
Yes – antibody testing		1.86(1.28-2.70)**	
Yes – unsure about either of the three	0.49(0.37-0.64)***		0.46(0.36-0.58)***
Presence of underlying diseases (SA)			
Yes	1.00		
No	1.07(0.99-1.16)		
Living with family members who are elderly or have underlying diseases (SA)			

Yes		1.00	1.00
No		0.88(0.79-0.98)*	0.87(0.80-0.94)***
Do you receive an influenza vaccine? (SA)			
Every year		1.00	
Every few years		0.90(0.81-1.00)*	
Rarely or never		0.73(0.67-0.80)***	
Did you receive on routine immunization? (SA)			
All	1.00	1.00	1.00
Partially	0.82(0.76-0.89)***	0.86(0.76-0.97)*	0.92(0.84-1.01)
None	0.81(0.75-0.87)***	0.98(0.88-1.09)	0.93(0.85-1.00)*
Not sure	0.75(0.69-0.81)***	0.95(0.85-1.07)	0.91(0.83-1.00)*
How anxious are you about COVID-19? (SA)			
Not at all anxious	1.00	1.00	1.00
Vaguely anxious	1.55(1.38-1.76)***	2.01(1.79-2.25)***	1.52(1.37-1.68)***
Have a clear sense of anxiety	2.58(2.26-2.94)***	2.68(2.34-3.07)***	1.68(1.49-1.89)***
Feel fear and have anxiety	3.26(2.79-3.82)***	2.20(1.84-2.62)***	1.76(1.51-2.05)***
To what extent did the COVID-19 pandemic affect your life, within the past year? (SA)			
Not at all	1.00	1.00	1.00
Not much	1.11(0.97-1.27)	1.28(1.12-1.45)***	1.26(1.12-1.42)***
Somewhat	1.30(1.14-1.49)***	2.08(1.83-2.37)***	1.61(1.43-1.81)***
Quite a lot	1.60(1.38-1.84)***	2.59(2.21-3.02)***	1.85(1.62-2.12)***
What is your best guess as to whether you will get COVID-19 within the next 6 months? (SA)			
I don't think I will get COVID-19	1.00	1.00	1.00
I think I will get a mild case of COVID-19	0.92(0.87-0.98)**	0.89(0.82-0.96)**	0.95(0.89-1.01)
I think I will get seriously ill from COVID-19	1.14(1.05-1.24)**	0.85(0.76-0.95)**	0.97(0.89-1.07)
I have already had COVID-19	0.21(0.12-0.35)***	0.29(0.20-0.41)***	0.37(0.26-0.52)***
Has anyone close to you ever been infected with COVID-19? (MA)			
Family or friends		0.77(0.64-0.93)**	
Colleagues at work	1.30(1.07-1.60)*	0.73(0.63-0.85)***	
No/I don't know.	1.36(1.15-1.60)***		1.19(1.08-1.32)**
Have you ever refrained from visiting a medical institution because you were anxious about being infected by the new coronavirus? (MA)			
Have refrained from regular hospital visits	1.54(1.43-1.66)***	1.22(1.01-1.47)*	1.34(1.17-1.54)***
Have refrained from visiting the hospital for sudden symptoms such as a cold, headache, or stomachache	1.22(1.12-1.34)***	1.35(1.11-1.64)**	1.52(1.32-1.75)***
Have refrained from medical checkups, physical examinations, or cancer screenings	1.36(1.25-1.48)***	1.64(1.36-1.99)***	1.34(1.17-1.54)***
Have refrained from other medical examinations	1.25(1.15-1.36)***	1.29(1.06-1.58)*	1.29(1.11-1.50)**
No		1.25(1.03-1.52)*	1.22(1.06-1.41)**
When a vaccine for COVID-19 becomes available, will you get vaccinated? (SA)			
Yes		1.00	1.00
No		0.88(0.77-1.01)	0.91(0.81-1.03)
Not sure		1.05(0.96-1.16)	1.07(0.99-1.16)
How do you feel are the benefits of the COVID-19 vaccine? (SA)			

Very small	1.00	1.00	1.00
Small	1.14(0.91-1.42)	1.18(0.93-1.50)	1.34(1.08-1.65)**
Medium	1.26(1.03-1.55)*	1.32(1.06-1.65)*	1.51(1.24-1.85)***
Large	1.27(1.04-1.57)*	1.65(1.32-2.07)***	1.63(1.33-1.99)***
Very large	1.33(1.07-1.67)*	1.64(1.28-2.10)***	1.60(1.29-1.99)***
How do you think the disadvantages of the COVID-19 vaccine are? (SA)			
Very small		1.00	1.00
Small		1.03(0.89-1.21)	0.97(0.86-1.10)
Medium		1.01(0.86-1.19)	0.98(0.86-1.11)
Large		0.92(0.77-1.09)	0.89(0.77-1.03)
Very large		0.93(0.73-1.17)	1.01(0.82-1.24)
If others have been vaccinated against COVID-19, do you think you should be vaccinated as well? (SA)			
Strongly disagree	1.00	1.00	1.00
Disagree	1.04(0.86-1.27)	0.97(0.78-1.20)	0.97(0.80-1.18)
Neither agree nor disagree	1.14(0.94-1.38)	0.90(0.73-1.12)	0.91(0.75-1.11)
Agree	1.11(0.92-1.35)	1.10(0.88-1.38)	1.03(0.85-1.26)
Strongly agree	1.36(1.10-1.68)**	1.06(0.82-1.37)	1.15(0.93-1.44)
Do you think getting a COVID-19 vaccine will ease your anxiety? (SA)			
Strongly disagree	1.00	1.00	1.00
Disagree	0.82(0.69-0.98)*	0.80(0.65-0.99)*	0.63(0.52-0.76)***
Neither agree nor disagree	0.80(0.67-0.95)**	0.63(0.51-0.78)***	0.56(0.46-0.67)***
Agree	0.72(0.60-0.86)***	0.62(0.50-0.78)***	0.55(0.45-0.66)***
Strongly agree	0.77(0.61-0.96)*	0.72(0.53-0.97)*	0.50(0.39-0.64)***
Do you trust scientists in the field of vaccine development for COVID-19? (SA)			
Strongly distrust			1.00
Distrust			1.35(0.99-1.84)
Neutral			1.31(0.96-1.78)
Trust			1.36(0.99-1.86)
Strongly trust			1.45(1.00-2.10)
Do you trust the public authorities to approve vaccines for COVID-19? (SA)			
Strongly distrust	1.00	1.00	1.00
Distrust	0.82(0.67-1.02)	0.85(0.67-1.09)	0.86(0.69-1.08)
Neutral	0.70(0.57-0.86)**	0.82(0.64-1.04)	0.77(0.62-0.97)*
Trust	0.74(0.60-0.91)**	0.80(0.62-1.04)	0.77(0.61-0.98)*
Strongly trust	0.72(0.55-0.96)*	0.46(0.32-0.66)***	0.61(0.45-0.84)**
Do you trust your healthcare provider about vaccination against COVID-19? (SA)			
Strongly distrust	1.00	1.00	1.00
Distrust	1.25(0.92-1.70)	1.21(0.88-1.66)	1.21(0.88-1.67)
Neutral	1.25(0.93-1.68)	1.30(0.96-1.76)	1.27(0.92-1.73)
Trust	1.22(0.90-1.65)	1.45(1.06-1.98)*	1.41(1.02-1.95)*
Strongly trust	1.08(0.78-1.50)	1.48(1.03-2.12)*	1.52(1.07-2.17)*
Do you think that healthcare workers and employees of elderly care facilities should be vaccinated? (SA)			

Yes	1.00	1.00	1.00
No	0.59(0.48-0.71)***	0.56(0.48-0.66)***	0.64(0.55-0.75)***
Can't say either	0.87(0.80-0.94)***	0.87(0.79-0.95)**	0.93(0.86-1.01)
Use of information source			
Healthcare professionals	1.36(1.26-1.47)***	1.27(1.13-1.43)***	1.27(1.16-1.39)***
TV, radio and newspaper	1.47(1.35-1.61)***	2.15(1.96-2.36)***	1.89(1.74-2.05)***
Scientific literature	1.36(1.07-1.72)*	1.48(1.02-2.16)*	
Books and magazines	1.15(1.03-1.28)*		1.26(1.10-1.44)**
The Internet	1.37(1.30-1.46)***	1.92(1.79-2.06)***	1.64(1.55-1.74)***
Medical information sites	1.31(1.10-1.55)**	2.16(1.57-2.97)***	1.39(1.12-1.72)**
Blogs or web pages of celebrities and famous people		1.34(0.97-1.86)	
Governments	1.42(1.34-1.5)***	1.92(1.74-2.11)***	1.73(1.61-1.85)***
Family and friends	1.12(1.05-1.20)**	1.52(1.36-1.69)***	1.34(1.24-1.45)***
Scientists and researchers	1.34(1.14-1.58)**		1.41(1.13-1.77)**
Pharmaceutical and other companies		0.82(0.68-0.98)*	0.83(0.70-0.98)*
Social media	1.12(1.04-1.2)**	1.44(1.30-1.60)***	1.48(1.36-1.61)***
Trust of information source			
Healthcare professionals	1.10(1.05-1.16)**	1.12(1.04-1.20)**	1.13(1.06-1.19)***
Books and magazines		0.88(0.82-0.95)***	0.92(0.86-0.98)**
The Internet	0.96(0.90-1.01)		1.06(1.00-1.12)
Medical information sites		1.09(1.02-1.16)*	1.11(1.05-1.17)***
Blogs or web pages of celebrities and famous people		0.90(0.84-0.98)**	0.91(0.86-0.97)**
Public sectors		1.15(1.06-1.24)**	
Family and friends		1.07(0.99-1.15)	
Scientists and researchers		1.13(1.04-1.22)**	1.11(1.05-1.17)***
Pharmaceutical and other companies	1.08(1.02-1.15)*	0.91(0.83-1.01)	
Social media	0.91(0.86-0.96)**	0.73(0.67-0.80)***	0.81(0.76-0.87)***

*: $p<0.05$, **: $p<0.01$, ***: $p<0.001$
OR: Odds Ratio, CI: Confidence Intervals.
3Cs: "closed spaces", "crowded places", and "close-contact settings"

Table S2. Number of information sources used and adoption of preventive measures**.**

	OR (95% CI)		
	3Cs	Hand hygiene	Respiratory hygiene
Number of information sources used			
1	1.00	1.00	1.00
2	1.39(1.28-1.52)***	1.92(1.76-2.10)***	1.68(1.55-1.81)***
3	1.62(1.48-1.78)***	2.70(2.43-2.99)***	2.16(1.99-2.35)***
4	1.92(1.74-2.11)***	3.65(3.21-4.14)***	2.83(2.57-3.13)***
5 or more	2.70(2.48-2.94)***	5.92(5.27-6.64)***	4.68(4.29-5.11)***
Age (SA)			
20's	1.00	1.00	1.00

30's	1.43(1.26-1.61)***	1.45(1.29-1.64)***	1.56(1.40-1.74)***
40's	1.60(1.42-1.81)***	1.45(1.28-1.63)***	1.83(1.64-2.04)***
50's	2.06(1.81-2.33)***	1.60(1.41-1.83)***	2.33(2.07-2.62)***
60's	2.16(1.90-2.46)***	1.59(1.39-1.81)***	2.37(2.10-2.68)***
70's+	2.17(1.89-2.49)***	1.56(1.34-1.80)***	2.02(1.77-2.31)***
Gender (SA)			
Female	1.00	1.00	1.00
Male	0.70(0.65-0.74)***	0.61(0.56-0.66)***	0.58(0.55-0.62)***
Highest educational level (SA)			
Middle and High school	1.00	1.00	1.00
Junior college	1.06(0.98-1.14)	1.08(0.98-1.19)	1.13(1.04-1.23)**
University	1.05(0.98-1.11)	0.94(0.87-1.02)	1.01(0.95-1.08)
Graduate School	1.14(0.99-1.30)	0.78(0.66-0.92)**	0.88(0.76-1.01)
Region (SA)			
Hokkaido and Tohoku	1.00	1.00	1.00
Kanto	1.37(1.26-1.50)***	1.11(0.99-1.25)	1.23(1.12-1.35)***
Chubu	1.04(0.95-1.15)	0.87(0.77-0.99)*	1.10(0.99-1.22)
Kinki	1.18(1.07-1.30)**	0.96(0.84-1.09)	1.17(1.05-1.29)**
Chugoku and Shikoku	1.03(0.92-1.16)	0.74(0.64-0.85)***	1.00(0.89-1.12)
Kyushu	1.09(0.98-1.22)	0.90(0.78-1.04)	1.21(1.08-1.36)**
Occupation type (SA)			
Agriculture, forestry and fisheries	1.00	1.00	1.00
Construction	1.18(0.81-1.73)	1.09(0.72-1.63)	1.37(0.96-1.96)
Manufacturing	1.24(0.87-1.76)	1.21(0.83-1.77)	1.4(1.01-1.96)*
Information and communications	1.18(0.81-1.72)	1.35(0.89-2.03)	1.55(1.09-2.22)*
Transportation and postal services	1.03(0.70-1.51)	1.56(1.03-2.35)*	1.45(1.01-2.07)*
Wholesale and retail trade	1.16(0.81-1.66)	1.37(0.92-2.02)	1.44(1.03-2.02)*
Finance and insurance	1.25(0.85-1.83)	1.38(0.90-2.12)	1.44(1.00-2.08)
Real estate and goods rental and leasing	1.01(0.67-1.52)	0.98(0.62-1.53)	1.35(0.91-1.99)
Scientific research, professional and technical services	1.42(0.94-2.13)	1.59(0.99-2.56)	1.66(1.12-2.48)*
Accommodations, food and beverage services	1.15(0.78-1.70)	1.25(0.81-1.92)	1.33(0.92-1.93)
Living-related and personal services and amusement services	1.08(0.73-1.62)	1.58(1.00-2.50)	1.36(0.93-2.00)
Education and learning support	1.12(0.78-1.63)	1.34(0.89-2.03)	1.43(1.00-2.04)
Medical healthcare and welfare	1.13(0.79-1.61)	1.19(0.81-1.77)	1.27(0.90-1.78)
Combined services	1.24(0.79-1.94)	1.11(0.67-1.83)	1.45(0.94-2.24)
Services (not elsewhere classified)	1.22(0.85-1.74)	1.34(0.91-1.97)	1.44(1.03-2.00)*
Public service (not elsewhere classified)	0.99(0.68-1.45)	1.25(0.83-1.88)	1.70(1.19-2.43)**
Students	0.96(0.63-1.45)	1.16(0.76-1.79)	1.11(0.76-1.61)
Homemaker	1.50(1.06-2.13)*	1.59(1.09-2.34)*	1.58(1.13-2.20)**
Others	1.29(0.91-1.83)	1.30(0.89-1.89)	1.18(0.85-1.63)
Household size including respondent (SA)			
1	1.00	1.00	1.00

2	1.19(1.09-1.31)***	1.27(1.15-1.39)***	1.19(1.08-1.30)**
3	1.06(0.97-1.17)	1.24(1.12-1.37)***	1.07(0.98-1.18)
4	0.97(0.87-1.08)	1.18(1.05-1.32)**	1.17(1.05-1.31)**
5	1.04(0.90-1.21)	1.37(1.15-1.62)**	1.28(1.10-1.49)**
More than 6	0.93(0.76-1.14)	1.07(0.84-1.36)	1.10(0.89-1.35)
Marital size (SA)			
Married (including de facto marriage)	1.00		1.00
Not married (without partner)	1.10(1.01-1.20)*		1.15(1.06-1.26)**
Not married (with a partner)	1.00(0.88-1.15)		1.15(1.01-1.31)
Widowed	1.02(0.89-1.17)		1.05(0.89-1.24)
Divorced	1.09(0.97-1.22)		1.17(1.03-1.33)*
Self-reported health status (SA)			
Very good	1.00		1.00
Good	0.91(0.85-0.98)**		1.02(0.95-1.10)
Fair	0.90(0.83-0.97)**		0.96(0.89-1.04)
Poor	0.88(0.80-0.98)*		0.94(0.85-1.05)
Very poor	0.97(0.81-1.18)		0.83(0.68-1.01)
How confident are you filling out medical forms by yourself?" (health literacy) (SA)			
Not at all	1.00	1.00	1.00
A little bit	0.91(0.69-1.20)	1.57(1.20-2.05)**	1.18(0.92-1.52)
Somewhat	1.08(0.83-1.39)	1.77(1.39-2.26)***	1.25(0.99-1.57)
Extremely	1.29(1.00-1.66)	2.39(1.88-3.04)***	1.53(1.22-1.93)***
Quite a bit	1.58(1.23-2.04)***	2.75(2.16-3.51)***	1.89(1.50-2.38)***
Have you ever received a COVID-19 test? (MA)			
No		2.43(1.96-3.01)***	
Yes – PCR test		1.95(1.53-2.48)***	
Yes – antigen test		1.57(1.12-2.18)**	0.71(0.55-0.91)**
Yes – antibody testing		1.89(1.31-2.72)**	
Yes – unsure about either of the three	0.48(0.36-0.63)***		0.42(0.34-0.53)***
Living with family members who are elderly or have underlying diseases (SA)			
Yes		1.00	1.00
No		0.88(0.79-0.97)*	0.87(0.80-0.94)***
Do you receive an influenza vaccine? (SA)			
Every year		1.00	
Every few years		0.90(0.81-1.00)*	
Rarely or never		0.74(0.67-0.81)***	
Did you receive on routine immunization? (SA)			
All	1.00	1.00	
Partially	0.81(0.75-0.88)***	0.85(0.76-0.96)**	
None	0.81(0.76-0.87)***	1.03(0.92-1.15)	
Not sure	0.74(0.68-0.81)***	0.97(0.86-1.09)	
How anxious are you about COVID-19? (SA)			

Not at all anxious	1.00	1.00	1.00
Vaguely anxious	1.56(1.38-1.76)***	2.08(1.86-2.32)***	1.56(1.40-1.73)***
Have a clear sense of anxiety	2.60(2.28-2.96)***	2.71(2.37-3.10)***	1.71(1.51-1.92)***
Feel fear and have anxiety	3.31(2.83-3.88)***	2.14(1.80-2.54)***	1.76(1.51-2.05)***
To what extent did the COVID-19 pandemic affect your life, within the past year? (SA)			
Not at all	1.00	1.00	1.00
Not much	1.10(0.96-1.27)	1.27(1.12-1.45)***	1.26(1.11-1.42)***
Somewhat	1.30(1.13-1.48)***	2.07(1.82-2.36)***	1.60(1.43-1.81)***
Quite a lot	1.59(1.38-1.83)***	2.57(2.20-3.00)***	1.86(1.62-2.13)***
What is your best guess as to whether you will get COVID-19 within the next 6 months? (SA)			
I don't think I will get COVID-19	1.00	1.00	1.00
I think I will get a mild case of COVID-19	0.92(0.86-0.98)**	0.87(0.81-0.94)***	0.94(0.88-1.00)*
I think I will get seriously ill from COVID-19	1.12(1.03-1.22)*	0.81(0.73-0.91)***	0.95(0.86-1.04)
I have already had COVID-19	0.21(0.13-0.35)***	0.23(0.17-0.32)***	0.32(0.22-0.44)***
Has anyone close to you ever been infected with COVID-19? (MA)			
Colleagues at work	1.31(1.08-1.60)**		
No/I don't know.	1.36(1.16-1.60)***	1.42(1.26-1.60)***	1.22(1.10-1.35)***
Have you ever refrained from visiting a medical institution because you were anxious about being infected by the new coronavirus? (MA)			
Have refrained from regular hospital visits	1.55(1.44-1.67)***	1.19(0.99-1.44)	1.35(1.18-1.55)***
Have refrained from visiting the hospital for sudden symptoms such as a cold, headache, or stomachache	1.24(1.13-1.35)***	1.33(1.10-1.60)**	1.54(1.34-1.78)***
Have refrained from medical checkups, physical examinations, or cancer screenings	1.36(1.26-1.48)***	1.64(1.36-1.98)***	1.36(1.18-1.56)***
Have refrained from other medical examinations	1.26(1.16-1.38)***	1.30(1.07-1.59)*	1.33(1.15-1.54)***
No		1.24(1.02-1.50)*	1.24(1.07-1.43)**
When a vaccine for COVID-19 becomes available, will you get vaccinated? (SA)			
Yes	1.00	1.00	1.00
No	0.91(0.80-1.03)	0.85(0.75-0.98)*	0.90(0.80-1.01)
Not sure	0.93(0.87-1.01)	1.06(0.96-1.16)	1.08(1.00-1.16)
How do you feel are the benefits of the COVID-19 vaccine? (SA)			
Very small	1.00	1.00	1.00
Small	1.17(0.94-1.45)	1.21(0.96-1.51)	1.33(1.08-1.64)*
Medium	1.31(1.06-1.61)*	1.39(1.13-1.72)**	1.53(1.25-1.87)***
Large	1.31(1.07-1.61)*	1.74(1.40-2.15)***	1.66(1.36-2.03)***
Very large	1.38(1.11-1.72)**	1.71(1.34-2.18)***	1.64(1.32-2.04)***
How do you think the disadvantages of the COVID-19 vaccine are? (SA)			
Very small			1.00
Small			0.98(0.87-1.11)
Medium			0.99(0.87-1.12)
Large			0.91(0.79-1.05)
Very large			0.97(0.79-1.20)
If others have been vaccinated against COVID-19, do you think you should be vaccinated as well? (SA)			
Strongly disagree	1.00	1.00	1.00

Disagree	1.03(0.85-1.26)	0.93(0.75-1.16)	0.94(0.78-1.14)
Neither agree nor disagree	1.13(0.92-1.37)	0.87(0.70-1.08)	0.88(0.73-1.07)
Agree	1.08(0.88-1.32)	1.08(0.86-1.35)	1.01(0.83-1.23)
Strongly agree	1.31(1.06-1.63)*	1.05(0.81-1.36)	1.16(0.93-1.44)
Do you think getting a COVID-19 vaccine will ease your anxiety? (SA)			
Strongly disagree	1.00	1.00	1.00
Disagree	0.83(0.70-0.99)*	0.82(0.66-1.01)	0.64(0.53-0.77)***
Neither agree nor disagree	0.80(0.67-0.95)**	0.64(0.52-0.79)***	0.56(0.46-0.67)***
Agree	0.71(0.60-0.85)***	0.64(0.52-0.80)***	0.55(0.45-0.66)***
Strongly agree	0.76(0.60-0.95)*	0.74(0.55-0.99)*	0.49(0.39-0.63)***
Do you trust scientists in the field of vaccine development for COVID-19? (SA)			
Strongly distrust			1.00
Distrust			1.36(1.00-1.85)
Neutral			1.35(1.00-1.84)
Trust			1.42(1.04-1.94)*
Strongly trust			1.47(1.01-2.12)*
Do you trust the public authorities to approve vaccines for COVID-19? (SA)			
Strongly distrust	1.00	1.00	1.00
Distrust	0.82(0.66-1.01)	0.86(0.67-1.09)	0.86(0.69-1.08)
Neutral	0.69(0.56-0.85)***	0.83(0.65-1.06)	0.77(0.61-0.96)*
Trust	0.73(0.59-0.90)**	0.80(0.62-1.03)	0.76(0.60-0.96)*
Strongly trust	0.71(0.53-0.93)*	0.43(0.30-0.60)***	0.59(0.43-0.81)**
Do you trust your healthcare provider about vaccination against COVID-19? (SA)			
Strongly distrust	1.00	1.00	1.00
Distrust	1.26(0.93-1.72)	1.25(0.92-1.71)	1.22(0.88-1.68)
Neutral	1.29(0.96-1.73)	1.39(1.03-1.87)*	1.29(0.95-1.77)
Trust	1.25(0.92-1.69)	1.54(1.13-2.10)**	1.44(1.05-1.98)*
Strongly trust	1.13(0.82-1.57)	1.60(1.12-2.30)*	1.60(1.12-2.27)*
Do you think that healthcare workers and employees of elderly care facilities should be vaccinated? (SA)			
Yes	1.00	1.00	1.00
No	0.57(0.47-0.70)***	0.52(0.44-0.60)***	0.60(0.51-0.70)***
Can't say either	0.88(0.81-0.95)**	0.85(0.78-0.93)***	0.92(0.85-0.99)*
Trust of information sources			
Healthcare professionals	1.11(1.05-1.17)***	1.12(1.04-1.20)**	1.14(1.08-1.21)***
TV, radio and newspaper	0.96(0.91-1.01)		
Books and magazines		0.86(0.80-0.93)***	0.93(0.88-0.99)*
Medical information sites		1.09(1.02-1.16)*	1.12(1.06-1.18)***
Blogs or web pages of celebrities and famous people		0.92(0.85-0.99)*	0.92(0.87-0.98)**
Public sectors		1.16(1.08-1.25)***	
Family and friends			0.95(0.90-1.00)*
Scientists and researchers		1.11(1.03-1.18)**	1.15(1.09-1.21)***
Pharmaceutical and other companies	1.09(1.03-1.15)**		

Social media	0.90(0.85-0.94)***	0.71(0.66-0.77)***	0.82(0.76-0.88)***

*: p<0.05, **: p<0.01, ***: p<0.001
OR: Odds Ratio, CI: Confidence Intervals.
3Cs: "closed spaces", "crowded places", and "close-contact settings"

Table S3. Number of social network services used and adoption of preventive measures**.**

	OR (95% CI)		
	3Cs avoidance	Hand hygiene	Respiratory hygiene
Number of social network services used			
0	1.00	1.00	1.00
1	1.24(1.15-1.35)***	1.76(1.57-1.96)***	1.74(1.59-1.90)***
2	1.44(1.23-1.68)***	1.78(1.45-2.20)***	1.96(1.65-2.33)***
3	1.76(1.34-2.31)***	1.80(1.26-2.57)**	1.88(1.40-2.53)***
4	2.41(1.59-3.65)***	2.19(1.27-3.78)**	3.23(1.99-5.24)***
5	2.68(1.53-4.70)**	4.86(1.85-12.79)**	2.32(1.22-4.40)*
6	4.55(2.03-10.19)***	3.28(1.03-10.47)*	9.35(2.64-33.13)**
Age (SA)			
20's	1.00	1.00	1.00
30's	1.53(1.36-1.73)***	1.62(1.44-1.83)***	1.72(1.55-1.92)***
40's	1.79(1.59-2.03)***	1.72(1.52-1.94)***	2.11(1.89-2.35)***
50's	2.34(2.06-2.66)***	2.00(1.74-2.29)***	2.74(2.44-3.09)***
60's	2.58(2.27-2.94)***	2.19(1.90-2.53)***	3.06(2.71-3.46)***
70's+	2.72(2.37-3.12)***	2.40(2.04-2.82)***	2.88(2.52-3.29)***
Gender (SA)			
Female	1.00	1.00	1.00
Male	0.70(0.66-0.75)***	0.65(0.61-0.71)***	0.61(0.57-0.65)***
Highest educational level (SA)			
Middle and High school	1.00	1.00	1.00
Junior college	1.08(1.00-1.16)	1.11(1.00-1.22)	1.15(1.07-1.25)**
University	1.07(1.01-1.14)*	0.97(0.89-1.05)	1.02(0.96-1.09)
Graduate School	1.16(1.02-1.34)*	0.81(0.69-0.95)**	0.88(0.77-1.01)
Region (SA)			
Hokkaido and Tohoku	1.00	1.00	1.00
Kanto	1.34(1.23-1.47)***	1.08(0.96-1.21)	1.17(1.07-1.29)**
Chubu	1.03(0.93-1.14)	0.88(0.78-1.00)*	1.08(0.97-1.19)
Kinki	1.17(1.06-1.29)**	0.96(0.85-1.09)	1.14(1.03-1.26)*
Chugoku and Shikoku	1.03(0.92-1.16)	0.76(0.66-0.87)***	1.00(0.89-1.12)
Kyushu	1.09(0.98-1.21)	0.91(0.80-1.05)	1.20(1.08-1.34)**
Occupation type (SA)			
Agriculture, forestry and fisheries	1.00	1.00	1.00
Construction	1.19(0.81-1.73)	1.06(0.71-1.58)	1.32(0.93-1.87)
Manufacturing	1.26(0.88-1.79)	1.20(0.83-1.75)	1.37(0.99-1.90)
Information and communications	1.18(0.81-1.72)	1.32(0.88-1.96)	1.47(1.04-2.09)*

Transportation and postal services	1.02(0.70-1.50)	1.50(1.00-2.24)	1.39(0.98-1.98)
Wholesale and retail trade	1.19(0.83-1.70)	1.40(0.96-2.05)	1.45(1.04-2.02)*
Finance and insurance	1.28(0.87-1.87)	1.37(0.90-2.07)	1.41(0.99-2.03)
Real estate and goods rental and leasing	1.01(0.67-1.51)	0.90(0.59-1.4)	1.25(0.85-1.83)
Scientific research, professional and technical services	1.49(1.00-2.24)	1.74(1.1-2.77)*	1.74(1.17-2.58)**
Accommodations, food and beverage services	1.17(0.80-1.72)	1.30(0.85-1.97)	1.33(0.92-1.91)
Living-related and personal services and amusement services	1.12(0.75-1.67)	1.54(0.99-2.41)	1.33(0.91-1.95)
Education and learning support	1.18(0.82-1.71)	1.43(0.96-2.15)	1.48(1.04-2.10)*
Medical healthcare and welfare	1.16(0.81-1.66)	1.23(0.84-1.81)	1.28(0.92-1.79)
Combined services	1.23(0.79-1.93)	1.10(0.68-1.80)	1.39(0.91-2.14)
Services (not elsewhere classified)	1.25(0.88-1.79)	1.37(0.95-2.00)	1.44(1.04-2.01)*
Public service (not elsewhere classified)	1.05(0.72-1.53)	1.33(0.89-1.99)	1.74(1.22-2.47)**
Students	0.97(0.64-1.48)	1.15(0.76-1.75)	1.11(0.77-1.61)
Homemaker	1.53(1.08-2.18)*	1.7(1.17-2.47)**	1.59(1.15-2.20)**
Others	1.29(0.91-1.83)	1.28(0.89-1.85)	1.16(0.84-1.60)
Annual household income in 2020 (million JPY) (SA)		1.02(1.00-1.03)	1.02(1.01-1.03)**
Household size including respondent (SA)			
1	1.00	1.00	1.00
2	1.24(1.14-1.36)***	1.40(1.25-1.56)***	1.22(1.12-1.34)***
3	1.11(1.01-1.22)*	1.36(1.22-1.52)***	1.10(1.00-1.21)
4	1.02(0.91-1.13)	1.32(1.16-1.50)***	1.20(1.07-1.33)**
5	1.09(0.94-1.27)	1.48(1.23-1.77)***	1.29(1.11-1.50)**
More than 6	0.98(0.80-1.20)	1.20(0.94-1.53)	1.12(0.91-1.38)
Marital size (SA)			
Married (including de facto marriage)	1.00	1.00	1.00
Not married (without partner)	1.14(1.04-1.24)**	1.19(1.07-1.32)**	1.23(1.12-1.34)***
Not married (with a partner)	1.05(0.91-1.20)	1.23(1.06-1.43)**	1.23(1.08-1.40)**
Widowed	1.02(0.89-1.17)	1.05(0.86-1.30)	1.07(0.91-1.25)
Divorced	1.08(0.97-1.21)	1.23(1.05-1.43)*	1.20(1.06-1.36)**
Self-reported health status (SA)			
Very good	1.00	1.00	1.00
Good	0.93(0.87-1.00)*	1.05(0.96-1.16)	1.05(0.97-1.13)
Fair	0.92(0.85-0.99)*	0.98(0.89-1.08)	0.99(0.91-1.07)
Poor	0.90(0.82-1.00)*	1.04(0.91-1.18)	0.97(0.88-1.08)
Very poor	0.99(0.82-1.19)	0.84(0.67-1.04)	0.84(0.69-1.02)
How confident are you filling out medical forms by yourself?" (health literacy) (SA)			
Not at all	1.00	1.00	1.00
A little bit	0.95(0.72-1.25)	1.57(1.20-2.04)**	1.22(0.96-1.57)
Somewhat	1.12(0.87-1.44)	1.78(1.41-2.27)***	1.31(1.05-1.64)*
Extremely	1.39(1.08-1.78)*	2.55(2.01-3.23)***	1.68(1.34-2.09)***
Quite a bit	1.74(1.35-2.23)***	3.03(2.39-3.86)***	2.11(1.68-2.64)***
Have you ever received a COVID-19 test? (MA)			

No		2.78(2.25-3.42)***	1.49(1.14-1.96)**
Yes – PCR test		2.26(1.78-2.86)***	1.41(1.06-1.88)*
Yes – antigen test		1.75(1.27-2.41)**	
Yes – antibody testing		2.12(1.49-3.03)***	1.51(1.07-2.12)*
Yes – unsure about either of the three	0.43(0.33-0.57)***		0.56(0.40-0.80)**
Living with family members who are elderly or have underlying diseases (SA)			
Yes		1.00	1.00
No		0.82(0.74-0.91)***	0.81(0.75-0.88)***
Do you receive an influenza vaccine? (SA)			
Every year	1.00	1.00	
Every few years	0.99(0.92-1.07)	0.92(0.83-1.02)	
Rarely or never	0.94(0.88-1.01)	0.74(0.67-0.81)***	
Did you receive on routine immunization? (SA)			
All	1.00	1.00	1.00
Partially	0.82(0.76-0.90)***	0.85(0.75-0.95)**	0.91(0.83-1.00)*
None	0.80(0.74-0.86)***	0.94(0.84-1.05)	0.88(0.82-0.96)**
Not sure	0.72(0.67-0.79)***	0.91(0.81-1.02)	0.87(0.80-0.95)**
How anxious are you about COVID-19? (SA)			
Not at all anxious	1.00	1.00	1.00
Vaguely anxious	1.62(1.44-1.83)***	2.24(2.01-2.49)***	1.67(1.51-1.85)***
Have a clear sense of anxiety	2.75(2.42-3.13)***	3.02(2.65-3.45)***	1.90(1.70-2.14)***
Feel fear and have anxiety	3.28(2.80-3.83)***	2.15(1.81-2.54)***	1.79(1.54-2.08)***
To what extent did the COVID-19 pandemic affect your life, within the past year? (SA)			
Not at all	1.00	1.00	1.00
Not much	1.18(1.03-1.35)*	1.41(1.24-1.59)***	1.38(1.22-1.55)***
Somewhat	1.46(1.28-1.67)***	2.47(2.18-2.81)***	1.89(1.68-2.12)***
Quite a lot	1.80(1.57-2.08)***	3.10(2.67-3.61)***	2.21(1.93-2.52)***
What is your best guess as to whether you will get COVID-19 within the next 6 months? (SA)			
I don't think I will get COVID-19	1.00	1.00	1.00
I think I will get a mild case of COVID-19	0.91(0.86-0.97)**	0.87(0.81-0.94)***	0.94(0.88-0.99)*
I think I will get seriously ill from COVID-19	1.09(1.00-1.18)	0.80(0.72-0.90)***	0.92(0.84-1.01)
I have already had COVID-19	0.18(0.11-0.30)***	0.19(0.14-0.26)***	0.27(0.19-0.37)***
Has anyone close to you ever been infected with COVID-19? (MA)			
Colleagues at work	1.28(1.05-1.56)*		
No/I don't know.	1.33(1.13-1.56)**	1.41(1.26-1.59)***	1.21(1.10-1.34)***
Have you ever refrained from visiting a medical institution because you were anxious about being infected by the new coronavirus? (MA)			
Have refrained from regular hospital visits	1.58(1.47-1.69)***	1.36(1.14-1.64)**	1.45(1.27-1.66)***
Have refrained from visiting the hospital for sudden symptoms such as a cold, headache, or stomachache	1.28(1.17-1.40)***	1.56(1.29-1.88)***	1.68(1.46-1.93)***
Have refrained from medical checkups, physical examinations, or cancer screenings	1.42(1.31-1.54)***	1.86(1.55-2.25)***	1.48(1.29-1.70)***
Have refrained from other medical examinations	1.30(1.19-1.41)***	1.45(1.19-1.77)***	1.42(1.23-1.64)***
No		1.35(1.12-1.63)**	1.28(1.11-1.47)**

When a vaccine for COVID-19 becomes available, will you get vaccinated? (SA)			
Yes	1.00	1.00	1.00
No	0.90(0.79-1.01)	0.83(0.72-0.94)**	0.87(0.77-0.98)*
Not sure	0.95(0.88-1.02)	1.08(0.99-1.18)	1.09(1.01-1.18)*
How do you feel are the benefits of the COVID-19 vaccine? (SA)			
Very small	1.00	1.00	1.00
Small	1.20(0.96-1.49)	1.27(1.02-1.58)*	1.36(1.11-1.68)**
Medium	1.32(1.07-1.61)**	1.43(1.17-1.75)**	1.54(1.26-1.87)***
Large	1.36(1.11-1.67)**	1.90(1.54-2.34)***	1.74(1.43-2.12)***
Very large	1.44(1.16-1.79)**	1.84(1.45-2.33)***	1.72(1.39-2.12)***
How do you think the disadvantages of the COVID-19 vaccine are? (SA)			
Very small			1.00
Small			0.97(0.87-1.10)
Medium			0.96(0.85-1.09)
Large			0.89(0.77-1.03)
Very large			0.91(0.74-1.12)
If others have been vaccinated against COVID-19, do you think you should be vaccinated as well? (SA)			
Strongly disagree	1.00	1.00	1.00
Disagree	1.03(0.84-1.25)	0.96(0.78-1.18)	0.96(0.79-1.15)
Neither agree nor disagree	1.09(0.90-1.33)	0.84(0.68-1.04)	0.86(0.71-1.04)
Agree	1.05(0.86-1.29)	1.07(0.86-1.33)	1.00(0.82-1.21)
Strongly agree	1.29(1.04-1.59)*	1.06(0.83-1.37)	1.14(0.92-1.41)
Do you think getting a COVID-19 vaccine will ease your anxiety? (SA)			
Strongly disagree	1.00	1.00	1.00
Disagree	0.83(0.70-0.99)*	0.83(0.67-1.01)	0.64(0.54-0.77)***
Neither agree nor disagree	0.77(0.65-0.92)**	0.60(0.49-0.74)***	0.53(0.45-0.64)***
Agree	0.70(0.59-0.83)***	0.62(0.50-0.76)***	0.53(0.44-0.64)***
Strongly agree	0.70(0.56-0.87)**	0.64(0.48-0.85)**	0.45(0.35-0.57)***
Do you trust scientists in the field of vaccine development for COVID-19? (SA)			
Strongly distrust			1.00
Distrust			1.39(1.03-1.88)*
Neutral			1.39(1.03-1.89)*
Trust			1.47(1.08-2.00)*
Strongly trust			1.49(1.04-2.15)*
Do you trust the public authorities to approve vaccines for COVID-19? (SA)			
Strongly distrust	1.00	1.00	1.00
Distrust	0.83(0.68-1.03)	0.84(0.66-1.07)	0.84(0.68-1.05)
Neutral	0.69(0.56-0.84)***	0.78(0.62-0.99)*	0.73(0.58-0.91)**
Trust	0.71(0.58-0.88)**	0.74(0.58-0.95)*	0.70(0.56-0.88)**
Strongly trust	0.67(0.50-0.88)**	0.37(0.26-0.52)***	0.51(0.37-0.70)***
Do you trust your healthcare provider about vaccination against COVID-19? (SA)			
Strongly distrust	1.00	1.00	1.00

Distrust	1.29(0.95-1.76)	1.28(0.95-1.74)	1.23(0.90-1.69)
Neutral	1.35(1.00-1.81)	1.49(1.11-2.00)**	1.36(1.00-1.85)
Trust	1.36(1.01-1.84)*	1.79(1.32-2.42)***	1.60(1.17-2.19)**
Strongly trust	1.24(0.90-1.72)	1.91(1.34-2.72)***	1.80(1.27-2.54)**
Do you think that healthcare workers and employees of elderly care facilities should be vaccinated? (SA)			
Yes	1.00	1.00	1.00
No	0.53(0.44-0.64)***	0.46(0.40-0.54)***	0.55(0.47-0.64)***
Can't say either	0.88(0.81-0.95)**	0.84(0.77-0.92)***	0.92(0.85-0.99)*
Trust in information sources			
Healthcare professionals	1.11(1.05-1.17)***	1.09(1.01-1.16)*	1.1(1.03-1.16)**
TV, radio and newspaper	0.94(0.88-0.99)*		
Books and magazines		0.88(0.82-0.95)***	0.92(0.87-0.98)**
Sientific literature			1.07(1.01-1.13)*
The Internet		1.08(1.00-1.17)	1.07(1.01-1.14)*
Medical information sites		1.15(1.08-1.23)***	1.15(1.09-1.21)***
Blogs or web pages of celebrities and famous people		0.89(0.82-0.96)**	0.90(0.85-0.96)**
Governments	1.08(1.02-1.14)*	1.28(1.19-1.38)***	1.12(1.06-1.19)***
Family and friends		1.07(0.99-1.15)	
Scientists and researchers	1.10(1.05-1.15)***	1.18(1.09-1.27)***	1.16(1.09-1.23)***
Pharmaceutical and other companies		0.89(0.81-0.98)*	0.94(0.87-1.01)
Social media	0.86(0.82-0.91)***	0.63(0.58-0.69)***	0.73(0.68-0.78)***

*: $p<0.05$, **: $p<0.01$, ***: $p<0.001$
OR: Odds Ratio, CI: Confidence Intervals.
3Cs: “closed spaces”, “crowded places”, and “close-contact settings”
